# Two New Diterpenoids from the Buds of *Wikstroemia chamaedaphne*

**DOI:** 10.3390/molecules17066424

**Published:** 2012-05-29

**Authors:** Jieru Guo, Jinwen Zhang, Penghua Shu, Lingmei Kong, Xincai Hao, Yongbo Xue, Zengwei Luo, Yan Li, Gao Li, Guangmin Yao, Yonghui Zhang

**Affiliations:** 1 Hubei Key Laboratory of Natural Medicinal Chemistry and Resource Evaluation, School of Pharmacy, Tongji Medical College, Huazhong University of Science and Technology, Wuhan 430030, China; 2 Tongji Hospital Affiliated to Tongji Medical College, Huazhong University of Science and Technology, Wuhan 430030, China; 3 State Key Laboratory of Phytochemistry and Plant Resources in West China, Kunming Institute of Botany, Chinese Academy of Science, Kunming 650204, China

**Keywords:** *Wikstroemia chamaedaphne*, diterpenoids, lignans, cytotoxicity

## Abstract

Two new diterpenoids, wikstroelide Q (**1**) and prostratin Q (**5**), together with three known diterpenoids, pimelea factors P_2_ (**2**), P_3_ (**3**), and prostratin (**4**), and five known lignans, (+)-epipioresinol (**6**), (+)-isolariciresinol (**7**), (−)-lariciresinol (**8**), (+)-*epi*-sesaminone (**9**), and prestegane B (**10**), were isolated from the buds of *Wikstroemia chamaedaphne* Meissn. Their structures were elucidated by a combination of spectroscopic analyses. Compounds **1**–**10** were evaluated for their cytotoxicities against HL-60, SMMC-7721, A549, MCF-7, SW480, and BEAS-2B cell lines *in vitro*.

## 1. Introduction

**Figure 1:**
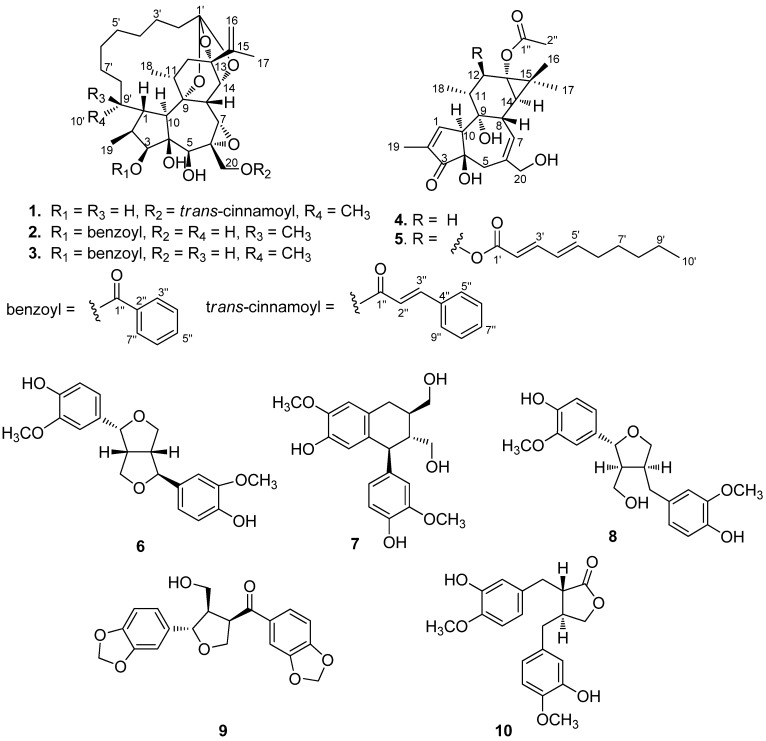
Structures of compounds **1**–**10**.

*Wikstroemia chamaedaphne* Meissn. (Thymelaeaceae), a toxic shrub endemic to China that has been used in folk medicine to treat edema, cough, hepatitis, schizophrenia, and antifertility [1,2]. Several flavonoids and the antifertile daphnane diterpenoid simplexin have been isolated from this medicinal plant in previous phytochemical investigations [2,3]. In the course of a search for novel anticancer natural products from Traditional Chinese Medicine, the acetone extract of the buds of *W. chamaedaphne* showed potential *in vitro* cytotoxic activities against HL-60, SMMC-7721, A549, MCF-7, and SW480 cell lines. Bioassay-guided isolation resulted in two new (compounds **1** and **5**) and three known diterpenoids **2**–**4**, along with five known lignans **6**–**10**. In this paper, we would like to report the isolation and structure elucidation of two new diterpenoids, named wikstroelide Q (**1**) and prostratin Q (**5**), and the cytotoxic activities of compounds **1**–**10** (Figure 1).

## 2. Results and Discussion

The acetone extract of the air-dried buds of *W. chamaedaphne* was partitioned successively with petroleum, CHCl_3_, and H_2_O as described in the Experimental. After repeated column chromatography, two new compounds **1** and **5** and three known diterpenoids **2**–**4**, together with five known lignans **6**–**10**, were isolated and identified. The known compounds were identified as pimelea factors P_2_ (**2**) [4,5], P_3_ (**3**) [5,6], prostratin (**4**) [7], (+)-epipioresinol (**6**) [8], (+)-isolariciresinol (**7**) [8], (−)-lariciresinol (**8**) [9], (+)-episesaminone (**9**) [10], and prestegane B (**10**) [11], respectively, on the basis of detailed MS and NMR spectroscopic analysis and comparison with those reported data in the literature.

Wikstroelide Q (**1**) was isolated as a white amorphous powder, [α]20.0 D +20.00 (*c* 0.22, MeOH). Its molecular formula C_39_H_52_O_9_ was assigned by the positive HRESIMS data (*m/z* 687.3486 [M+Na]^+^, calcd for C_39_H_52_O_9_Na, 687.3504), requiring 14 degrees of unsaturation. Its IR absorptions revealed the presence of hydroxyls (3441 cm^−^^1^), carbonyl (1713 cm^−^^1^), double bond (1637 cm^−^^1^), and benzene ring (1604 cm^−1^). The ^1^H-NMR spectrum of **1** (Table 1) showed signals for four methyl groups at *δ*_H_ 1.75 (3H, s), 1.25 (3H, d, *J* = 7.0 Hz), 1.04 (3H, d, *J* = 6.6 Hz), and 0.89 (3H, d, *J* =7.3 Hz), seven protons attached to oxygenated carbons at *δ*_H_4.93 (1H, d, *J* = 11.9 Hz), 4.23 (1H, d, *J* = 2.4 Hz), 3.90 (1H, d, *J* = 11.9 Hz), 3.79 (1H, d, *J* = 2.0 Hz), 3.73 (1H, br s), 3.30 (1H, s), and 3.01 (1H, d, *J* = 2.4 Hz), two olefinic protons at *δ*_H_7.72 (1H, d, *J* = 16.0 Hz) and 6.52 (1H, d, *J* = 16.0 Hz), and a mono-substituted benzene ring at *δ*_H_7.52 (2H, m) and 7.37 (3H, m). The ^13^C-NMR, DEPT, and HSQC spectra for **1** displayed thirty nine carbon signals differentiated as four methyls, ten methylenes (including one oxygenated and one olefinic), seventeen methines (including seven olefins, four oxygenated), and eight quaternary carbons (including one carbonyl, one olefin, and four oxygenated). 

**Figure 2:**
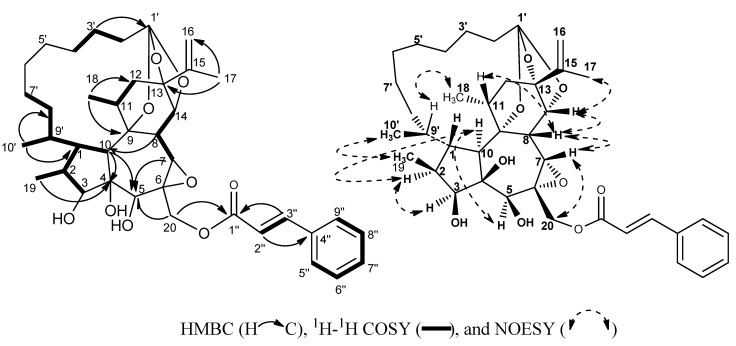
Key HMBC, ^1^H-^1^H COSY and NOESY of compound **1**.

The NMR data of **1** were quite similar to those of pimelea factor P_3_ (**3**) [5,6], a 1α-alkyldaphnane-type diterpenoid orthoester with a ten-carbon side chain, with the exception of the additional signals for one double bond (*δ*_H_ 6.52, d, *J=* 16.0 Hz, H-2′′; *δ*_H_ 7.72 d, *J=* 16.0 Hz, H-3′′;*δ*_C_ 117.9, C-2′′; *δ*_C_ 145.8, C-3′′) in the downfield region of the spectra. In the HMBC spectrum, the cross peaks of these two newly olefinic protons H-2′′ and H-3′′ to the conjugated carbonyl C-1′′ (*δ*_C_ 167.5) and the aromatic carbon C-4′′ (*δ*_C_ 135.3) of the mono-substituted benzene ring, as well as the aromatic protons H-5′′/H-9′′of the mono-substituted benzene ring to the newly olefinic carbon C-3′′, revealed that this additional double bond was fixed between the carbonyl and the benzene ring, and suggested the presence of a cinnamoyl group in **1**. The larger coupling constants *J*_H-2′′, H-3′′_ = 16.0 Hz of H-2′′ and H-3′′ indicated the *E*-geometry of the double bond in the cinnamoyl group, and the cinnamoyl group is the *trans*-cinnamoyl group. 

**Table 1:** .^1^H-NMR (400 MHz) and ^13^C-NMR (100 MHz) Spectral Data of Compounds **1** and **5** in CDCl_3_ (*δ* in ppm, *J* in Hz).

No.	1		5	
	*δ*_H_	*δ*_C_	*δ*_H_	*δ*_C_
1β	2.02 dd (12.4, 11.8)	49.3	7.57 s	161.0
2α	1.57 m	37.9		133.1
3α	3.79 d (2.0)	79.5		209.1
4		78.9		74.0
5α	3.73 br s	71.0	2.53 d (19.0)	38.9
5β			2.46 d (19.0)	
6		60.2		140.7
7β	3.30 s	64.4	5.66 d (4.5)	129.5
8β	3.01 d (2.4)	37.0	3.22 dd (4.5, 5.1)	39.3
9		81.4		78.4
10α	2.77 d (12.4)	48.8	3.23 s	56.4
11	2.36 m	35.8	2.15 m overlap	43.4
12a12b	2.17 dd (8.4, 13.9) 1.66 d (13.9)	36.6	5.44 d (10.3)	76.8
13		84.1		66.0
14	4.23 d (2.4)	82.1	1.08 d (5.1)	36.6
15		147.0		26.0
16a16b	4.97 s4.86 s	111.2	1.24s	17.0
17	1.75 s	19.1	1.19 s	24.0
18	1.25 d (7.0)	21.3	0.87 d (6.6)	14.6
19	1.04 d (6.6)	14.8	1.75 d (1.5)	10.3
20a20b	4.93 d (11.9) 3.90 d (11.9)	68.1	4.02 d (13.0)3.97 d (13.0)	68.3
1′		120.0		167.3
2′	1.89 m	33.9	5.76 d (15.4)	119.1
3′	1.53 m1.66 m	19.7	7.21 dd (15.4, 9.8)	145.8
4′	1.22 m	27.8	6.16 dd (9.8, 15.2)	128.5
5′	1.23 m	24.2	6.13 m	145.5
6′	1.30 m	24.7	2.13 m overlap	33.2
7′	1.53 m1.39 m	24.3	1.41 m	28.6
8′	1.30 m0.94 m	24.5	1.28 m	31.6
9′	2.32 m	27.3	1.28 m	22.7
10′	0.89 d (7.3)	19.1	0.86 t (6.9)	14.2
1′′		167.3		174.0
2′′	6.52 d (16.0)	117.9	2.08 s	21.3
3′′	7.72 d (16.0)	145.8		
4′′		134.6		
5′′	7.52 m	128.4		
6′′	7.37 m	129.1		
7′′	7.37 m	130.7		
8′′	7.37 m	129.1		
9′′	7.52 m	128.4		

The HMBC correlation of H-20 (*δ*_H_ 4.93, d, *J =* 11.9 Hz, H-20a; *δ*_H_ 3.90, d, *J =* 11.9 Hz, H-20b) to the carbonyl C-1′′ of the *trans*-cinnamoyl group suggested the *trans*-cinnamoyl group was connected to 20-OH. HSQC, ^1^H–^1^H COSY, and HMBC analysis (Figure 2) allowed us to construct the planar structure of compound **1**. The relative configuration of compound **1** was determined by the coupling constants and the NOESY analysis (Figure 2). Similar to pimelea factor P_3_ (**3**) and other C_30_ 1α-alkyldaphnane-type diterpenoid orthoesters, the linkage between the penta-/sept- and sept-/hexa- rings in compound **1** are *trans*, and H-10 was randomly assigned in an *α*-orientation. The larger coupling constant *J* = 12.4 Hz of H-10*α* with H-1 indicated a *trans*-relationship between H-10*α* and H-1, and H-1was in a *β*-orientation, consequently, C-1 side chain had an α-orientation. The NOESY correlations of H-10*α* to H-2 and H-5 and H-2 to H-3 revealed that H-2 H-3, and H-5 were *α*-oriented. The singlet peak of H-7 and the smaller coupling constants *J* = 2.4 Hz of H-8 with H-14 revealed the *syn*-relationships of H-7, H-8, and H-14. The NOESY correlations of H-7/H-8, H-7/H-20b, H-8/H-11, and H-8/H-14 indicated that H-7, H-8, H-11, H-14 and H-20b were assigned in *β*-orientations. A literature survey revealed that when the absolute configuration of C-9′ was *R*, correspondingly, 10′-CH_3_ was in the *α*-orientation of the molecule, the chemical shift of C-10′ would appear around *δ*_C_ 19.0 [12,13]; if the C-9′ was *S* configuration, the C-10′ would shift downfield to *δ*_C_ 12.3 [4,5,13]. The chemical shift of C-10′ *δ*_C_ 19.1 in **1** suggested the *R* configuration of C-9′ in **1**. The NOESY correlations of CH_3_-10′ with CH_3_-19, H-1 and H-2, H-9′ with CH_3_-18 and H-1, and H-8′ with H-10, further supported the C-9′*R* configuration. Therefore, the structure of **1** was assigned as depicted. The *trans*-cinnamoyl group is very common in phenolic compounds, however, it was very rare in diterpenoids. To the best of our knowledge, compound **1** is the first example of C_30_ 1α-alkyldaphnane-type diterpenoid orthoester bearing a *trans*-cinnamoyl group.

Prostratin Q (**5**) was isolated as colorless gum, [α]20.0 D +16.36 (*c* 0.03, MeOH), and exhibited an quasi-molecular ion peak at *m/z* 579.2937 [M+Na]^+^ (calcd for C_32_H_44_O_8_Na, 579.2928), corresponding to the molecular formula C_32_H_44_O_8_. Its IR absorptions indicated the presence of hydroxyls (3420 cm^−1^), carbonyl (1714 cm^−1^), and double bond (1641 cm^−1^). The NMR spectral data (Table 1) of **5** were very similar to those of prostratin (**4**) [7], a phorbol-type diterpenoid isolated as a major compound in this study, except for an additional long chain aliphatic ester of C_10_H_15_O_2_. The NMR data of **5** indicated that the ester chain contained four olefinic protons at *δ*_H_ 5.76 (1H, d, *J* = 15.4 Hz, H-2′), 6.13 (1H, m, H-5′), 6.16 (1H, dd, *J* = 9.8, 15.2 Hz, H-4′), and 7.21 (1H, dd, = 15.4, 9.8 Hz, H-3′), with corresponding carbons at *δ*_C_ 119.1, 145.5, 128.5, 145.8, respectively. The ^1^H-^1^H COSY correlations of H-2′ to H-3′ (*δ*_H_ 7.21), H-3′ to H-4′, and H-4′ to H-5′, as well as HMBC correlations of H-2′ (*δ*_H_ 5.76) and H-3′ (*δ*_H_ 7.21) to the ester carbonyl C-1′ (*δ*_C_ 167.3) demonstrated that the ester carbonyl C-1′ and these two double bonds were conjugated. Additionally, the geometry of the Δ^2′^ and Δ^4′^ olefins in **5** was established as *Z* on the basis of the larger (*J*_H-2′, H-3′_ = 15.4 Hz, *J*_H-4′, H-5′_ = 15.2 Hz) coupling constants of H-2′ and H-4′. The HMBC correlation of H-12 (*δ*_H_ 5.44) to C-1′ (*δ*_C_ 167.3) indicated the ester chain was located at C-12 of the phorbol skeleton. The relative configurations of **5** were identical with those of prostratin (**4**), based on the detailed comparison of their coupling constants and the NOESY analysis. The larger coupling constants *J*_H-11, H-12_ = 10.3 Hz of H-12 and the significant NOSEY correlation between H-12 and CH_3_-18 permitted the assignment of ester chain substituent in the *β*-configuration.

Compounds **1**–**10** were evaluated for their cytotoxic activities against five human cancer cell lines, HL-60 (human myeloid leukemia), SMMC-7721 (hepatocellular carcinoma), A549 (lung cancer), MCF-7 (breast cancer), and SW480 (colon cancer), and one human normal cell line BEAS-2B (human bronchial epithelial) by the MTT method [14]. DDP (*cis*-platin) and taxol were used as positive controls. 

**Table 2:** IC_50_ Values (μM) of Compounds **1**–**10** against Five Human Cancer Cell Lines and One Human Normal Cell line.

Compounds	HL-60	SMMC-7721	A-549	MCF-7	SW480	BEAS-2B
1	26.43	>40	>40	>40	>40	>40
2	13.81	17.51	12.06	12.78	15.93	17.35
3	13.29	14.93	11.10	13.98	14.44	15.43
4	15.57	18.09	12.57	15.97	13.30	17.38
5	15.79	15.12	15.75	14.96	14.79	16.55
6	>40	>40	>40	>40	>40	>40
7	>40	>40	>40	>40	>40	>40
8	>40	>40	>40	>40	>40	>40
9	>40	>40	>40	>40	>40	>40
10	>40	>40	>40	>40	>40	>40
DDP ( *cis*-platin)	1.25	16.18	14.05	16.95	18.05	8.61
Taxol	<0.008	<0.008	<0.008	<0.008	<0.008	5.00

The bioassay results (Table 2) revealed that compound **1** exhibited weak cytotoxic activity against HL-60 cell lines with IC_50_ values of 26.43 μM, but was inactive against the SMMC-7721, A-549, MCF-7, SW480, and BEAS-2B cell lines (IC_50_ > 40 μM). Compounds **2**–**4** showed moderate cytotoxic activities [15] against the five human cancer cell lines and the human normal cell BEAS-2Bwithin the IC_50_ value range of 13–18 μM. Compounds **5**–**10** were inactive against the cancer cells used (IC_50_ > 40 μM). 

## 3. Experimental

### 3.1. General Procedures

Optical rotations were measured on a PerkinElmer PE-341LC polarimeter. IR spectra were recorded as KBr disks on a Bruker Vertex 70 FT-IR spectrophotometer. NMR spectra were recorded on a Bruker AM-400 spectrometer, and the ^1^H- and ^13^C-NMR chemical shifts were referenced to the solvent peaks for CDCl_3_ at *δ*_H_ 7.24 and *δ*_C_ 77.23. HRESIMS data were measured using an API QSTAR Pulsar spectrometer. Column chromatography was performed using silica gel (200–300 mesh, Qingdao Marine Chemical Inc., China), Amberchrom CG161M (75 *μ*m, Rohm and Haas, USA), ODS (50 *μ*m, YMC, Japan), and Sephadex LH-20 (Pharmacia Biotech AB, Sweden). HPLC separation was performed on an instrument consisting of an Agilent 1100 controller, an Agilent 1100 pump, and an Agilent UV detector with an YMC (250 × 10 mm, 5 μm) preparative column. TLC was carried out on precoated silica gel GF_254_ plates. Spots were visualized under UV light (254 or 356 nm) or by spraying with 5% H_2_SO_4_ in 95% EtOH followed by heating.

### 3.2. Plant Material

The buds of *W. chamaedaphne* were collected from Ankang City in Shaanxi Province, China, in July 2010, and authenticated by Prof. Changgong Zhang at School of Pharmacy, Tongji Medical College, Huazhong University of Technology and Science. The voucher specimen (No. TJ-1002) was deposited in the herbarium of Hubei Key Laboratory of Natural Medicinal Chemistry and Resource Evaluation, Tongji Medical College, Huazhong University of Technology and Science. 

### 3.3. Extraction and Isolation

The air-dried buds of *W. chamaedaphne* (40 kg) were extracted three times with acetone at room temperature (100 L each time, seven days). The combined acetone extracts were concentrated to yield a dry residue (1.9 Kg). This crude extract was suspended in H_2_O (4.0 L) and partitioned successively with petroleum ether (60–90 °C) and chloroform. The chloroform fraction (180 g) was chromatographed on silica gel (2 kg, 10.0 × 80 cm) using petroleum ether (60–90 °C)–ethyl acetate system to yield nine fractions. Fr. 3 (3.58 g) was subjected to an Amberchrom GC161M column eluted with EtOH/H_2_O, (3:2 to 9:1, v/v) to afford two fractions A and B. Fraction A (2.12) was chromatographed on ODS eluted with MeOH/H_2_O (4:6, v/v), followed by purification over semipreparative HPLC (45% MeOH in H_2_O, flow rate 2.0 ml/min, wavelength 210nm) to yield compounds **6** (8.0 mg, retention time 35 min) and **7** (7.6 mg, retention time 21 min). Fraction B (0.74 g) was subjected to Sephadex LH-20 (eluted with MeOH) and ODS column chromatography (eluted with 80% MeOH in H_2_O), followed by purification over semipreparative HPLC (92% MeOH in H_2_O, flow rate 2.0 ml/min, wavelength 210 nm) to yield **4** (15.0 mg, retention time 27 min) and **5** (12.0 mg, retention time 32 min). Fraction 4 (3.37 g) was fractionated by Amberchrom GC161M column (EtOH/H_2_O, 3:2 to 9:1, v/v) to afford Fractions C and D. Fraction C (1.76 g) was purified on a Sephadex LH-20 (eluted with MeOH) and a ODS column (MeOH/H_2_O 5:5) to give **10** (4.5 mg). Fraction D (0.87 g) was subjected into a silica gel column eluted with petroleum/EtOAc (5:1 to 1:2, v/v) to afford six major fractions, D1−D6. Fraction D2 was subjected to Sephadex LH-20 column chromatography (eluted with MeOH) to obtain the major portion, which was purified by a semipreparative HPLC (92% MeOH in H_2_O, flow rate 2.0 mL/min, wavelength 210 nm) to yield compounds **2** (15.0 mg, retention time 31 min) and **3** (9.8 mg, retention time 35 min). Fraction D3 was treated similarly to afford **1** (4.5 mg, retention time 33 min). Fr. 5 (12.37 g) was chromatographed over Amberchrom GC161M column (EtOH/H_2_O, 3:2 to 9:1, v/v) to afford two fractions E and F. Fraction E (7.56 g) was chromatographed over a silica gel column, eluted with petroleum ether (60–90°C)–ethyl acetate (4:1 to 1:1), to afford four major fractions, E1–E4. Fraction E2 (0.78 g) was purified by semipreparative HPLC (55% MeOH in H_2_O, flow rate 2.0 ml/min, wavelength 210 nm) to afford compound **9** (10.3 mg, retention time 23 min). Using the same procedure, Fraction E3 gave **8** (8.5 mg, retention time 27 min).

Wikstroelide Q (**1**): white amorphous powder; [α]20.0 D +20.00 (*c* 0.22, MeOH). UV (MeOH) *λ*_max_ (log *ε*) 275 (4.15) nm; IR (KBr)*ν*_max_ 3441, 2930, 1713, 1637, 1604, 1453, 1388, 1281, 1172, 1114, 1073, 1034,and 921 cm^−^^1^; ^1^H-NMR (CDCl_3_, 400 MHz) and ^13^C NMR (CDCl_3_, 100 MHz) see Table 1; HRESIMS *m*/*z* 687.3486 [M+Na]^+^ (calcd for C_39_H_52_O_9_Na, 687.3504).

ProstratinQ (**5**): colorless gum; [α]20.0 D +16.36 (*c* 0.03, MeOH). UV (MeOH) *λ*_max_ (log *ε*) 263 (3.84) nm; IR (KBr) *ν*_max_ 3420,2927, 1714, 1641, 1460, 1377, 1328, 1262, 1132, 1078, and 999 cm^−^^1^; ^1^H-NMR (CDCl_3_, 400 MHz) and ^13^C-NMR (CDCl_3_, 100 MHz) see Table 1; HRESIMS *m*/*z* 579.2937 [M + Na]^+^ (calcd for C_32_H_44_O_8_Na, 579.2928).

### 3.4. Cytotoxicity Assays

Five human cancer cell lines, human myeloid leukemia HL-60, hepatocellular carcinoma SMMC-7721, lung cancer A549, breast cancer MCF-7, and colon cancer SW480 cells, together with one human normal cell line BEAS-2B (human bronchial epithelial), were assayed. Cells were cultured in RPMI-1640 or in DMEM medium (Hyclone, USA), supplemented with 10% fetal bovine serum (Hyclone, USA) in 5% CO_2_ at 37 °C. The antiproliferative assay was performed according to the MTT (3-(4,5-dimethylthiazol-2-yl)-2,5-diphenyl tetrazolium bromide) method in 96-well microplates, as reported previously, with slight modification. [14] Briefly, 100 μL of adherent cells was seeded into each well of 96-well cell culture plates and allowed to adhere for 12 h before addition of test compounds, while suspended cells were seeded just before the addition of the drug with initial density of 1 × 10^5^ cells/mL. Each cancer cell line was exposed to the tested compound at concentrations of 0.0625, 0.32, 1.6, 8, and 40 μM in triplicates for 48 h. Wells with DMSO were used as negative controls, and DDP (*cis*-platin, Sigma, USA) and taxol were used as a positive control. After compound treatment, cell viability was detected by a Bio-Rad 680 at λ = 595 nm and a cell growth curve was graphed. IC_50_ values were calculated by Reed and Muench’s method. 

## 4. Conclusions

In conclusion, two new diterpenoids, wikstroelide Q (**1**) and prostratin Q (**5**), together with three known diterpenoids, pimelea factors P_2_ (**2**), P_3_ (**3**), and prostratin (**4**), and five known lignans, (+)–epipioresinol (**6**), (+)–isolariciresinol (**7**), (–)–lariciresinol (**8**), (+)–episesaminone (**9**), and prestegane B (**10**), were isolated from the buds of *Wikstroemia chamaedaphne* Meissn. Their structures were elucidated by a combination of spectroscopic analyses. Compound **1** exhibited weak cytotoxic activity against HL-60 cell lines with an IC_50_ value of 26.43 μM, but showed no active against SMMC-7721, A549, MCF-7, SW480 and BEAS-2B cell lines *in vitro*(IC_50_ > 40 μM). Compounds **2**–**4** showed moderate cytotoxic activities against HL-60, SMMC-7721, A549, MCF-7, SW480 and BEAS-2B cell lines within the IC_50_ value range of 13–18 μM. While compounds **5**–**10** were inactive (IC_50_ > 40 μM). 
